# Investigating the evolution and predicting the future outlook of antimicrobial resistance in sub-saharan Africa using phenotypic data for *Klebsiella pneumoniae*: a 12-year analysis

**DOI:** 10.1186/s12866-023-02966-y

**Published:** 2023-08-08

**Authors:** Dickson Aruhomukama, Hellen Nakabuye

**Affiliations:** Department of Medical Microbiology, College of Health Sciences, Makerere University, Kampala, Uganda

**Keywords:** Antimicrobial resistance, Evolution, Future outlook, Africa

## Abstract

**Background:**

Antimicrobial resistance (AMR) is a major public health challenge, particularly in sub-Saharan Africa (SSA). This study aimed to investigate the evolution and predict the future outlook of AMR in SSA over a 12-year period. By analysing the trends and patterns of AMR, the study sought to enhance our understanding of this pressing issue in the region and provide valuable insights for effective interventions and control measures to mitigate the impact of AMR on public health in SSA.

**Results:**

The study found that general medicine patients had the highest proportion of samples with AMR. Different types of samples showed varying levels of AMR. Across the studied locations, the highest resistance was consistently observed against ceftaroline (ranging from 68 to 84%), while the lowest resistance was consistently observed against ceftazidime avibactam, imipenem, meropenem, and meropenem vaborbactam (ranging from 92 to 93%). Notably, the predictive analysis showed a significant increasing trend in resistance to amoxicillin-clavulanate, cefepime, ceftazidime, ceftaroline, imipenem, meropenem, piperacillin-tazobactam, and aztreonam over time.

**Conclusions:**

These findings suggest the need for coordinated efforts and interventions to control and prevent the spread of AMR in SSA. Targeted surveillance based on local resistance patterns, sample types, and patient populations is crucial for effective monitoring and control of AMR. The study also highlights the urgent need for action, including judicious use of antibiotics and the development of alternative treatment options to combat the growing problem of AMR in SSA.

## Background

Antimicrobial resistance (AMR) is a growing public health concern worldwide, and it is particularly acute in sub-Saharan Africa (SSA), where infections caused by resistant bacteria are prevalent and pose a significant threat to health systems. The increasing prevalence of AMR in SSA is driven by several factors, including poor antimicrobial stewardship and limited access to effective diagnostics and therapeutics [1–4].

*Klebsiella pneumoniae* is a Gram-negative bacterium that is among the leading causes of healthcare-associated infections in SSA. *Klebsiella pneumoniae* can cause a range of infections, including pneumonia, urinary tract infections, bloodstream infections, and surgical site infections. However, its utility extends beyond its clinical significance, as it can serve as a proxy for other bacteria in studying AMR in SSA. One reason is that *Klebsiella pneumoniae* is commonly isolated from clinical specimens in the region, making it a useful indicator of the prevalence and patterns of AMR. Additionally, the bacterium has been associated with high rates of multidrug resistance, making it an ideal target for studying the evolution and spread of resistance in the region. Furthermore, *Klebsiella pneumoniae* has been found to share common resistance mechanisms with other Gram-negative bacteria, such as *Escherichia coli*, making it a potential proxy for studying resistance trends in other bacterial species [5–12].

Despite the growing threat of AMR in SSA, there is a scarcity of data on the evolution and future outlook of AMR in the region. This knowledge gap hampers the development of effective public health strategies and interventions to combat this escalating threat. Therefore, this study focused on *Klebsiella pneumoniae* as a proxy to provide valuable insights into the evolution and future outlook of AMR in SSA. By analysing phenotypic data for *Klebsiella pneumoniae* over a 12-year period, this research aimed to shed light on the patterns and potential trajectory of AMR in the region. The findings of this study have the potential to inform evidence-based interventions and policies to mitigate the impact of AMR on public health in SSA.

## Results

### Speciality

In Nigeria, the majority of samples in 2014, 2018, and 2020 were from general medicine (35 patients, 38%), general surgery (19 patients, 30%), and general medicine (54 patients, 41%), respectively. In South Africa, the majority of samples in 2014, 2018, and 2020 were from general medicine (37 patients, 37%; 33 patients, 39%; and 60 patients, 40%, respectively) (Table 1).

**Table 1:** Speciality for Nigeria and South Africa

Nigeria						South Africa					
2014		2018		2020		2014		2018		2020	
Speciality	Count	Speciality	Count	Speciality	Count	Speciality	Count	Speciality	Count	Speciality	Count
**Emergency Room**	6	**Emergency Room**	3	**Emergency Room**	13	**Emergency Room**	2	**Emergency Room**	1	**Emergency Room**	12
**General Unspecified ICU**	1	**Medicine General**	12	**Medicine General**	54	**General Unspecified ICU**	14	**General Unspecified ICU**	12	**General Unspecified ICU**	30
**Medicine General**	35	**Medicine ICU**	3	**Medicine ICU**	6	**Medicine General**	37	**Medicine General**	33	**Medicine General**	60
**Medicine ICU**	2	**None Given**	3	**None Given**	7	**Medicine ICU**	15	**Medicine ICU**	13	**Medicine ICU**	22
**None Given**	3	**Other**	3	**Other**	2	**None Given**	2	**Other**	5	**None Given**	4
**Pediatric General**	6	**Pediatric General**	7	**Pediatric General**	11	**Pediatric General**	6	**Pediatric General**	6	**Other**	1
**Pediatric ICU**	3	**Pediatric ICU**	10	**Pediatric ICU**	15	**Pediatric ICU**	7	**Pediatric ICU**	5	**Pediatric General**	5
**Surgery General**	31	**Surgery General**	19	**Surgery General**	21	**Surgery General**	10	**Surgery General**	8	**Pediatric ICU**	6
**Surgery ICU**	6	**Surgery ICU**	4	**Surgery ICU**	4	**Surgery ICU**	6	**Surgery ICU**	1	**Surgery General**	3
										**Surgery ICU**	6

### Source

In Nigeria, the majority of isolates in 2014, 2018, and 2020 were from urine (26 samples, 28%), blood (20 samples, 31%), and urine (56 samples, 42%), respectively. In South Africa, the majority of isolates in 2014, 2018, and 2020 were from wounds (22 samples, 22%), blood (25 samples, 30%), and blood (55 samples, 37%), respectively (Table 2).

**Table 2:** Source for Nigeria and South Africa

Nigeria						South Africa					
2014		2018		2020		2014		2018		2020	
Source	Count	Source	Count	Source	Count	Source	Count	Source	Count	Source	Count
**Abscess**	5	**Blood**	20	**Abscess**	1	**Abscess**	6	**Abscess**	2	**Abscess**	1
**Bladder**	1	**Colon**	2	**Blood**	27	**Bladder**	3	**Blood**	25	**Blood**	55
**Blood**	11	**Endotracheal aspirate**	2	**Burn**	2	**Blood**	1	**Bronchus**	1	**Bronchoalveolar lavage**	1
**Bronchoalveolar lavage**	1	**Gastric Abscess**	1	**Cellulitis**	2	**Bronchoalveolar lavage**	1	**Colon**	1	**Cellulitis**	1
**Burn**	3	**Genitourinary: Other**	2	**Decubitus**	3	**Colon**	3	**Endotracheal aspirate**	9	**Endotracheal aspirate**	25
**Cellulitis**	1	**Impetiginous lesions**	1	**Gastric Abscess**	1	**Endotracheal aspirate**	17	**Gastric Abscess**	4	**Gall Bladder**	3
**Endotracheal aspirate**	3	**Intestinal: Other**	1	**Sputum**	8	**Gastric Abscess**	2	**Intestinal: Other**	4	**Gastric Abscess**	4
**Gastric Abscess**	1	**None Given**	1	**Ulcer**	3	**Peritoneal Fluid**	6	**Liver**	1	**Impetiginous lesions**	1
**Impetiginous lesions**	1	**Prostate**	1	**Urethra**	2	**Sputum**	20	**None Given**	1	**Intestinal: Other**	3
**Peritoneal Fluid**	1	**Sputum**	1	**Urine**	56	**Urine**	18	**Peritoneal Fluid**	2	**Peritoneal Fluid**	13
**Prostate**	3	**Urethra**	4	**Wound**	28	**Wound**	22	**Skin: Other**	1	**Sputum**	22
**Sputum**	10	**Urine**	11					**Sputum**	13	**Stomach**	3
**Ulcer**	6	**Wound**	17					**Urine**	12	**Urine**	6
**Urethra**	1							**Wound**	8	**Wound**	11
**Urine**	26										
**Wound**	19										

### Antimicrobial susceptibility profiles

**Fig. 1:**
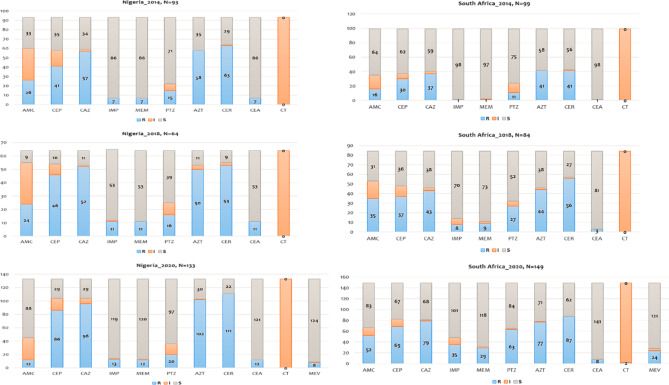
Antimicrobial susceptibility profiles for isolates from both Nigeria and South Africa

In Nigeria, the highest resistance in 2014 was to CER (63 isolates, 68%), while the lowest was to CEA, IMP, and MEM (86 isolates, 92% each). In 2018, the highest resistance was to CER, while the lowest was to CEA, IMP, and MEM (53 isolates, 83% each). In 2020, the highest resistance was to CER (111 isolates, 84%), while the lowest was to MEV (124 isolates, 93%). In South Africa, the highest resistance in 2014 was to CER (41 isolates, 41%), while the lowest was to CEA (98 isolates, 99%). In 2018, the highest resistance was to CER (51 isolates, 67%), while the lowest was to CEA (81 isolates, 96%). In 2020, the highest resistance was to CER (87 isolates, 58%), while the lowest was to MEV (121 isolates, 81%) (Fig. 1).

### Resistance patterns of bacterial isolates

The figure shows the highest and lowest resistance levels of isolates to selected antibiotics in each year. The bar charts illustrate three sections within each bar: the topmost section represents “S,” the middle section represents “I,” and the bottom section represents “R.” R- Resistant, I- Intermediate, S- Sensitive.

### Evolution

The isolates from Nigeria showed a significant increase in resistance to AMC, CEP, CAZ, AZT, and CER, while isolates from South Africa showed a significant increase in resistance to AMC, CEP, IMP, MEM, PTZ, and CER (Table 3).

**Table 3:** Evolution of AMR based on data from the countries

Nigeria (N2014 = 93, N2018 = 64, N2020 = 133)						South Africa (N2014 = 99, N2018 = 84, N2020 = 149)					
Year / Antibiotic	2014 n (%)	2018 n (%)	2020 n (%)	Chi^2^ Value	P-value	Year / Antibiotic	2014 n (%)	2018 n (%)	2020 n (%)	Chi^2^ Value	P-value
**AMC**	26 (28)	24 (38)	12 (9)	55.4397	**< 0.001**	**AMC**	16 (16)	35 (42)	52 (35)	23.9903	**< 0.001**
**CEP**	41 (44)	46 (72)	86 (65)	15.5951	**0.004**	**CEP**	30 (30)	37 (44)	69 (46)	10.6474	**0.031**
**CAZ**	57 (62)	52 (81)	96 (72)	12.3482	**0.015**	**CAZ**	37 (37)	43 (51)	79 (53)	7.4113	0.116
**IMP**	7 (6)	11 (17)	13 (10)	5.4172	0.247	**IMP**	1 (1)	8 (10)	35 (23)	39.3900	**< 0.001**
**MEM**	7 (6)	11 (17)	12 (9)	8.4687	0.076	**MEM**	1 (1)	9 (11)	29 (19)	20.3787	**< 0.001**
**PTZ**	15 (16)	16 (25)	20 (15)	6.5414	0.162	**PTZ**	11 (11)	27 (32)	63 (42)	36.3937	**< 0.001**
**AZT**	58 (62)	50 (78)	102 (77)	15.9586	**0.003**	**AZT**	41 (41)	44 (53)	77 (52)	6.5800	0.160
**CER**	63 (68)	53 (83)	111 (84)	13.4382	**0.009**	**CER**	41 (41)	56 (67)	87 (58)	14.9169	**0.005**
**CEA**	7 (6)	11 (17)	12 (9)	4.2779	0.118	**CEA**	1 (1)	3 (4)	8 (5)	3.2447	0.197
**CT**	0 (0)	0 (0)	0 (0)	-	-	**CT**	0 (0)	0 (0)	2 (1)	-	-
**MEV**	nt	nt	8 (6)	-	-	**MEV**	nt	nt	24 (16)	-	-

### Prediction to 2026

**Fig. 2:**
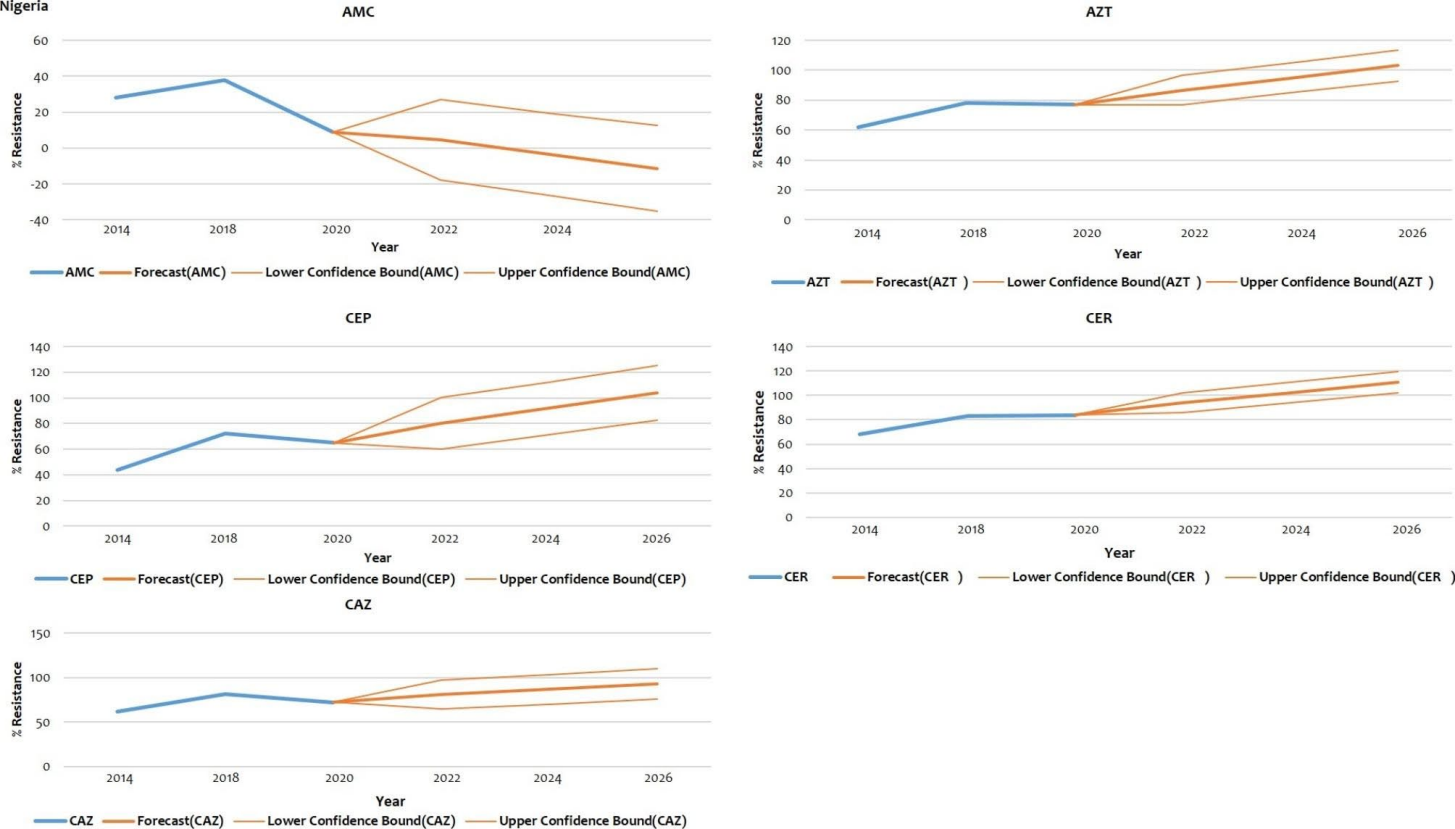
Prediction of AMR based on significant evolution data (Nigeria)

**Fig. 3:**
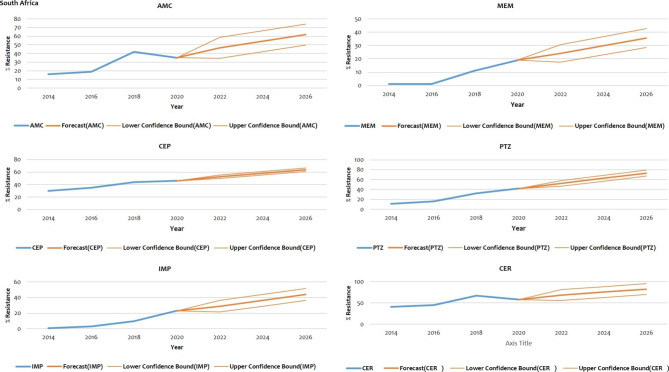
Prediction of AMR based on significant evolution data (South Africa)

The projections indicate a statistically significant likelihood of an increased trend in resistance towards antibiotics highlighted in Sect. 1.3.4 in both Nigeria and South Africa (Figs. 2 and 3 respectively).

### Resistance prediction for Nigeria

The bold blue lines represents resistance trends up to 2020. The bold red lines (in the middle) represent the projected resistance trends up to 2026. The non-bold red lines represent the lower and upper confidence bounds.

### Resistance prediction for South Africa

The bold blue lines represents resistance trends up to 2020. The bold red lines (in the middle) represent the projected resistance trends up to 2026. The non-bold red lines represent the lower and upper confidence bounds.

## Discussion

### Speciality

Based on the results, the majority of samples were from general medicine in both Nigeria and South Africa. This distribution is in agreement with findings from other studies conducted in SSA, implying that targeted surveillance of AMR in general medicine patients could be an effective approach to monitoring and controlling AMR in the region. By focusing on the most common patient population, resources can be used more efficiently, and interventions can be more targeted to where they are most needed. Nonetheless, it is worth noting that similar distribution of patients across specialties has been observed in other studies, underlining the significance of collaboration between different medical specialties and a comprehensive approach to tackling AMR in healthcare settings [13–18].

### Source

Based on the results, there is no consistent pattern in the types of samples from which isolates were most commonly obtained in both Nigeria and South Africa. This suggests that targeted surveillance of AMR should be based on the local epidemiology of infections and the sample types most commonly associated with AMR in each setting. For instance, if bloodstream infections are a significant problem in a particular hospital or region, then surveillance efforts should focus on blood isolates to monitor trends in AMR. Similarly, if urinary tract infections are more prevalent, then urine samples may be more useful for surveillance purposes. It is important to note that different sample types may have varying levels of AMR, and therefore, the selection of samples for surveillance should be guided by local data and expert opinion. Additionally, the use of standardized methods for specimen collection, transport, and processing is critical for ensuring the accuracy and comparability of surveillance data across different settings [19–25].

### Antimicrobial susceptibility profiles

Based on the results, there are significant differences in the resistance patterns of the isolates between Nigeria and South Africa. This suggests that targeted surveillance of AMR should be tailored to the local resistance patterns in a given setting, rather than a one-size-fits-all approach. For example, in Nigeria, CER had consistently high levels of resistance over the years, indicating the need for targeted surveillance and control measures for this antibiotic. In contrast, in South Africa, CER had varying levels of resistance over the years, and in 2020, the highest resistance was to a different antibiotic (MEV). This highlights the importance of regular surveillance to detect changes in resistance patterns and to guide appropriate interventions. It is also important to note that resistance patterns may vary not only between countries but also between hospitals, regions, and even different patient populations within the same hospital. Therefore, targeted surveillance efforts should be based on local data and guided by expert opinion to ensure that interventions are effective and appropriately targeted [19–28].

### Evolution and prediction

The results indicate a statistically significant increased trend in resistance towards several antibiotics in both Nigeria and South Africa. This highlights the urgent need for targeted surveillance of AMR in these countries, particularly for the antibiotics showing an increased trend in resistance. By monitoring the prevalence of AMR in specific antibiotics, healthcare providers can tailor their interventions to ensure optimal use of antibiotics and minimize the development and spread of resistant organism [29, 30].

It is important to note that the increase in resistance towards certain antibiotics may be attributed to various factors, such as antibiotic use patterns, prescribing practices, and patient demographics. Therefore, the implementation of effective targeted surveillance strategies should be coupled with other interventions, including antibiotic stewardship programs, infection control measures, and public awareness campaigns [5, 31–35].

Furthermore, understanding the classes of antibiotics that are becoming more resistant can provide insight into the mechanisms driving the development of AMR. This can inform research and development efforts aimed at creating new antibiotics and identifying alternative treatment options [36–40]. Ultimately, targeted surveillance of AMR can contribute to the development of evidence-based strategies to combat the rising threat of AMR in Nigeria, South Africa, and other countries in SSA.

### Limitation

Generalizability: While the results provide valuable insights into the situation in Nigeria and South Africa, caution should be exercised in generalizing the findings to other countries in SSA. The epidemiology of AMR can vary significantly across different regions and healthcare settings within SSA. Therefore, it is crucial to collect data from other countries in SSA to obtain a more comprehensive understanding of the patterns and drivers of AMR in the region. Data scope: This study focused on analysing data collected by the ATLAS programme (PFIZER) from Nigeria and South Africa over a specific period. While these countries were chosen due to the availability of complete data, it is important to acknowledge that they may not represent the entire spectrum of AMR dynamics within SSA. Including data from a larger number of countries within the region would contribute to a more representative assessment of AMR trends and enable better generalisation of the findings. Methodological considerations: The retrospective nature of the study and the use of phenotypic data analysis have inherent limitations. Molecular characterisation of resistance mechanisms and genomic analyses were not performed, which could provide additional insights into the genetic basis of resistance and the spread of resistant strains. Further studies could incorporate advanced molecular techniques to enhance the understanding of the mechanisms driving AMR in *Klebsiella pneumoniae* and other relevant pathogens. Longitudinal analysis: The study analysed data over a 12-year period, which provides valuable information on temporal trends in AMR. However, a longer-term longitudinal analysis covering an extended period would offer a more comprehensive understanding of the evolutionary dynamics of resistance and the impact of interventions over time. Additionally, such a study would be better suited to capture the emergence and spread of novel resistance mechanisms. Multidisciplinary collaboration: Although this study focused on the analysis of phenotypic data, collaboration with experts from diverse fields such as molecular biology, epidemiology, and healthcare policy could provide additional perspectives and enrich the study’s findings. Integrating multidisciplinary approaches could enable a more comprehensive assessment of AMR and facilitate the translation of research findings into effective interventions and policies.

## Conclusions

Overall, this study highlights the urgent need for action to address the growing problem of AMR in SSA, and the importance of a multifaceted approach that includes targeted surveillance, judicious use of antibiotics, and the development of alternative treatment options. The findings underscore the significance of tailoring interventions based on local resistance patterns, sample types, and patient populations. By implementing evidence-based strategies informed by comprehensive surveillance data, healthcare systems can effectively mitigate the impact of AMR and safeguard public health in SSA.

## Materials and methods

This study retrospectively analysed data collected by the ATLAS Programme (Pfizer). This programme’s aim is to do AMR surveillance across 80 countries, including 6 countries in SSA (Kenya, Nigeria, South Africa, Namibia, Cameroon, and Ivory Coast). This study targeted datasets for the 6 countries for the years 2014 to 2020. However, only 2 countries (Nigeria and South Africa) had complete data for these years hence were considered in the analysis. Only speciality, source, antimicrobial susceptibility profiles were extracted from the datasets. Antibiotic data targeted was for aztreonam (AZT), a monobactam; amoxycillin clavulanate (AMC) and piperacillin tazobactam (PTZ) both penicillin-β-lactam inhibitor combinations; ceftazidime (CAZ), a 3rd generation cephalosporin; ceftazidime avibactam (CEA), a 3rd generation cephalosporin-β-lactam inhibitor combination; cefepime (CEP), a 4th generation cephalosporin; ceftaroline (CER), a 5th generation cephalosporin; imipenem (IMP) and meropenem (MEM) both carbapenems; meropenem vaborbactam (MEV), a carbapenem-β-lactam inhibitor combination; and colistin (CT), a polypeptide. Prediction was done up to the year 2026.

## Data Availability

The data that support the findings of this study are available from https://amr.vivli.org but restrictions apply to the availability of these data, which were used under license for the current study, and so are not publicly available. Data are however available from the authors upon reasonable request and with permission of https://amr.vivli.org.

## Abbreviations

AMR: Antimicrobial resistance
AMC: Amoxycillin clavulanate
AZT: Aztreonam
CAZ: Ceftazidime
CEA: Ceftazidime avibactam
CEP: Cefepime
CT: Colistin
CER: Ceftaroline
PTZ: Piperacillin tazobactam
IMP: Imipenem
MEM: Meropenem
MEV: Meropenem vaborbactam
SSA: Sub-Saharan Africa
